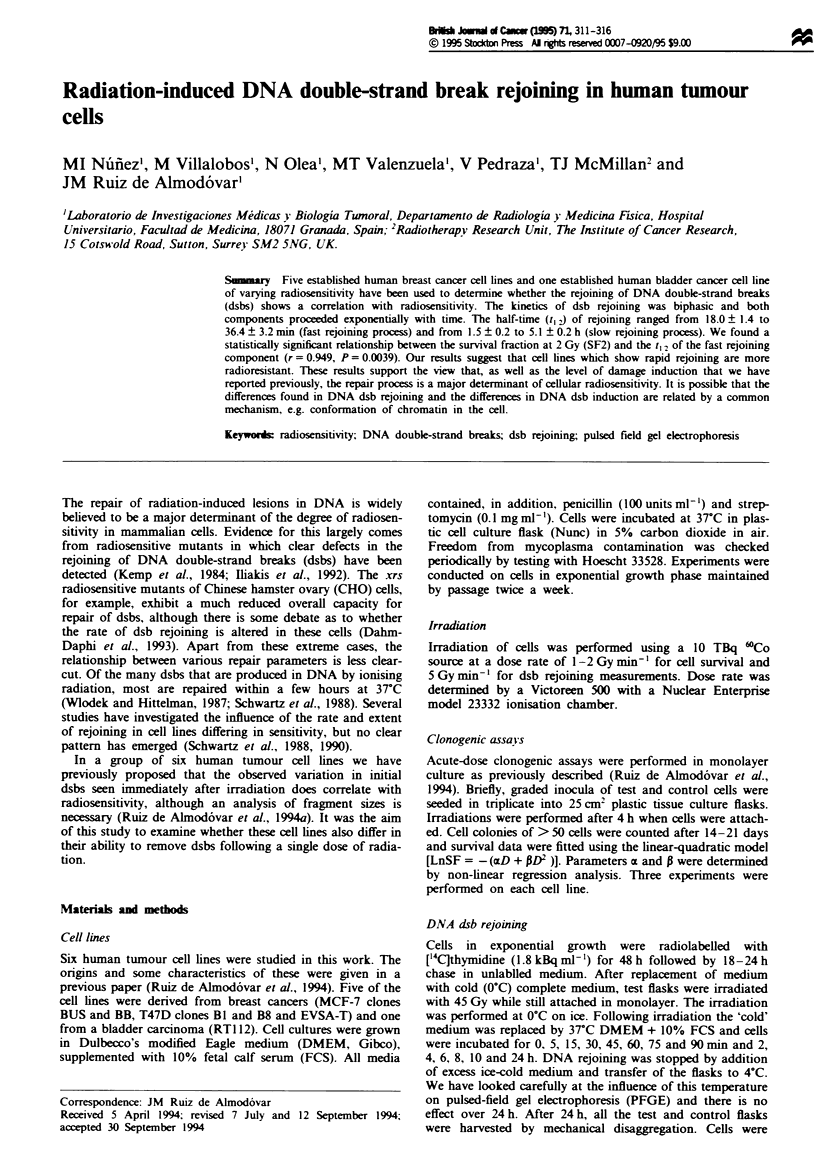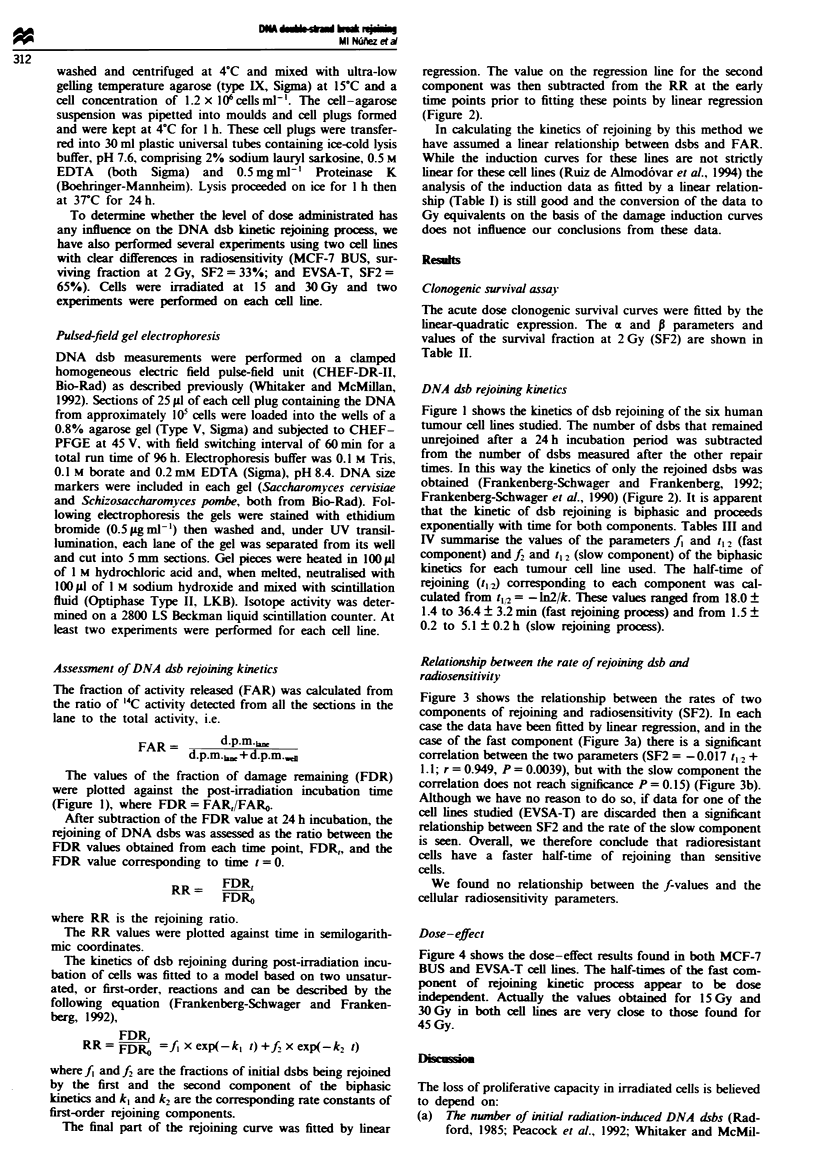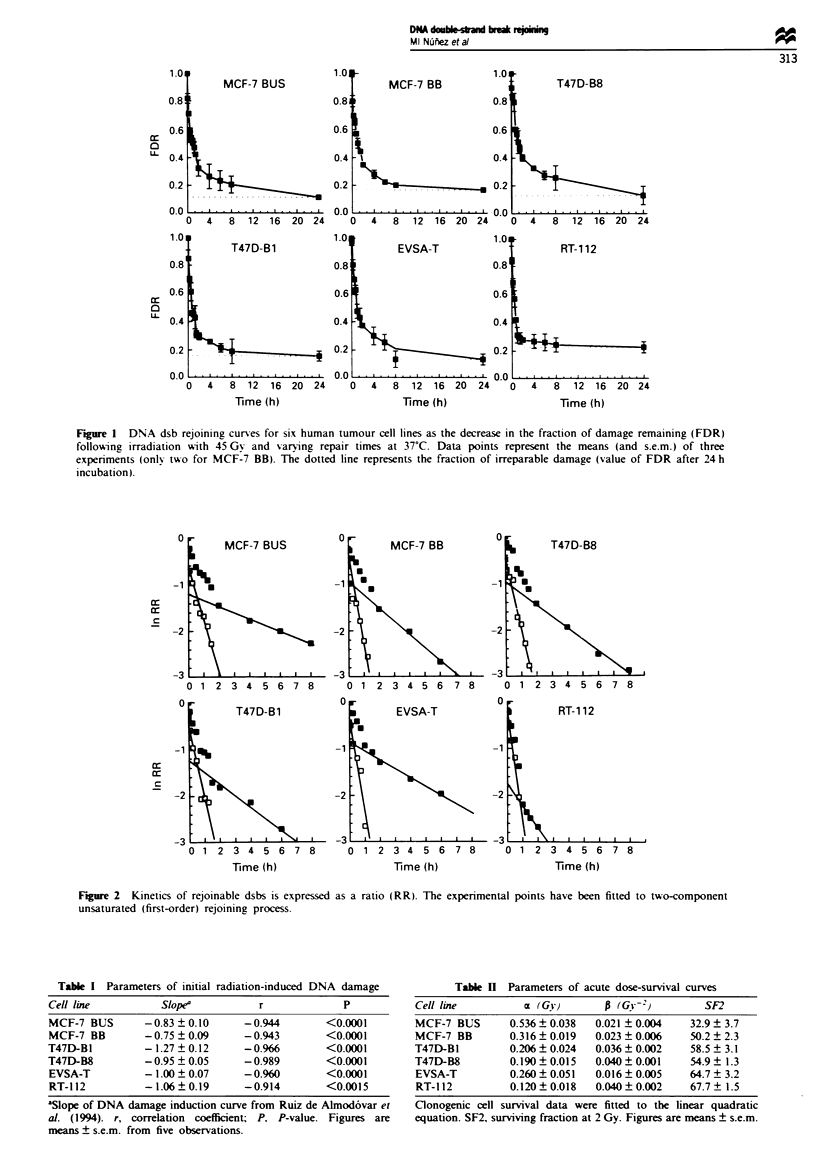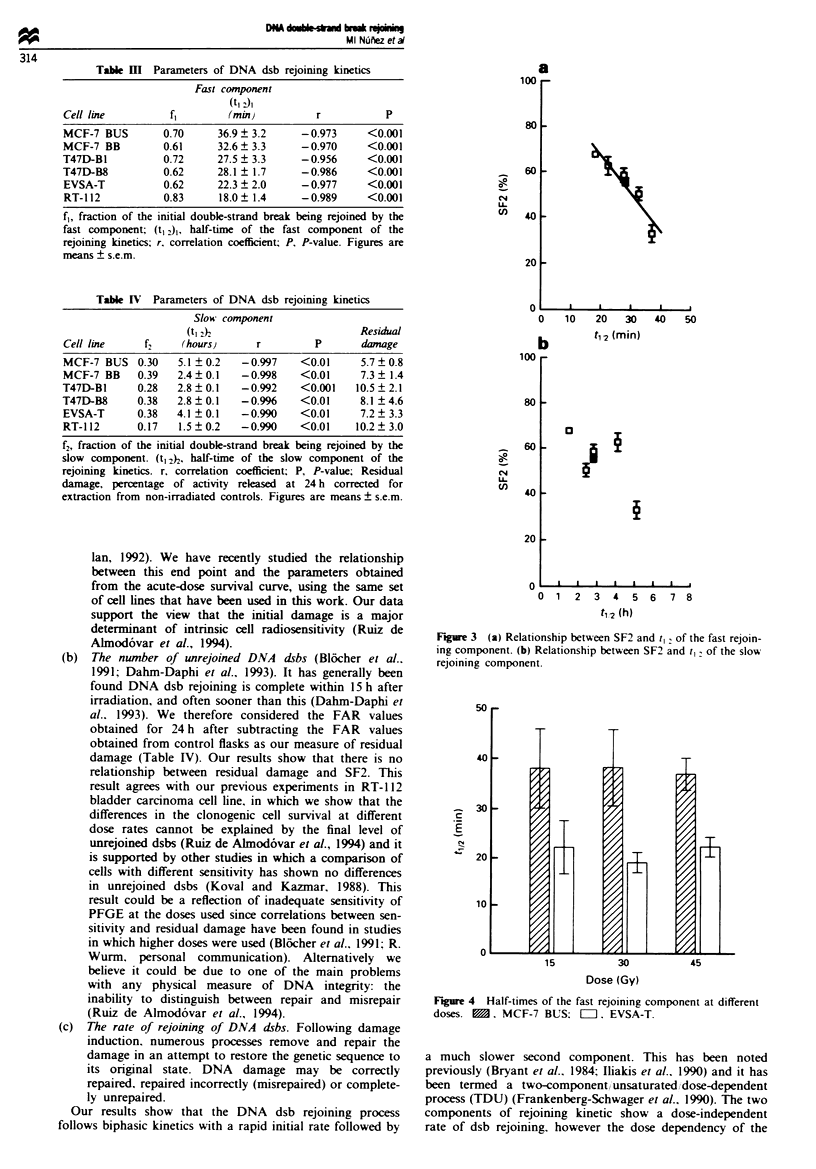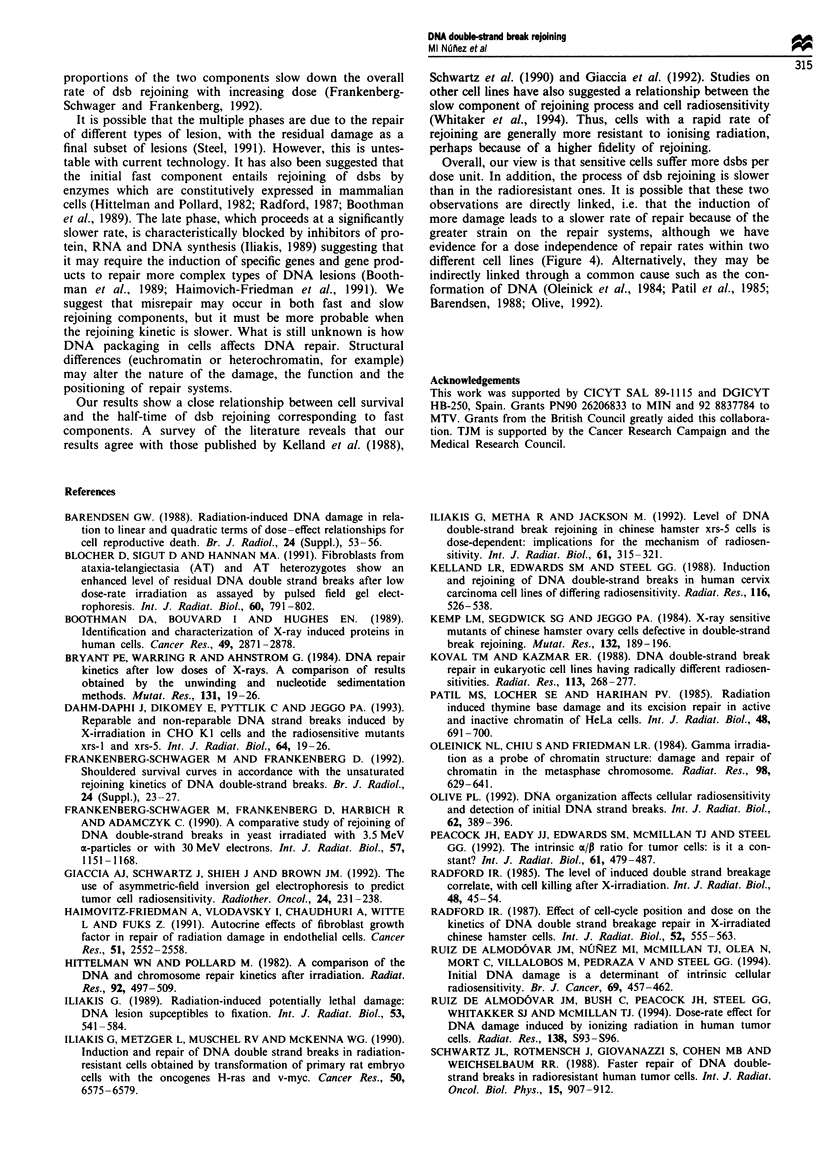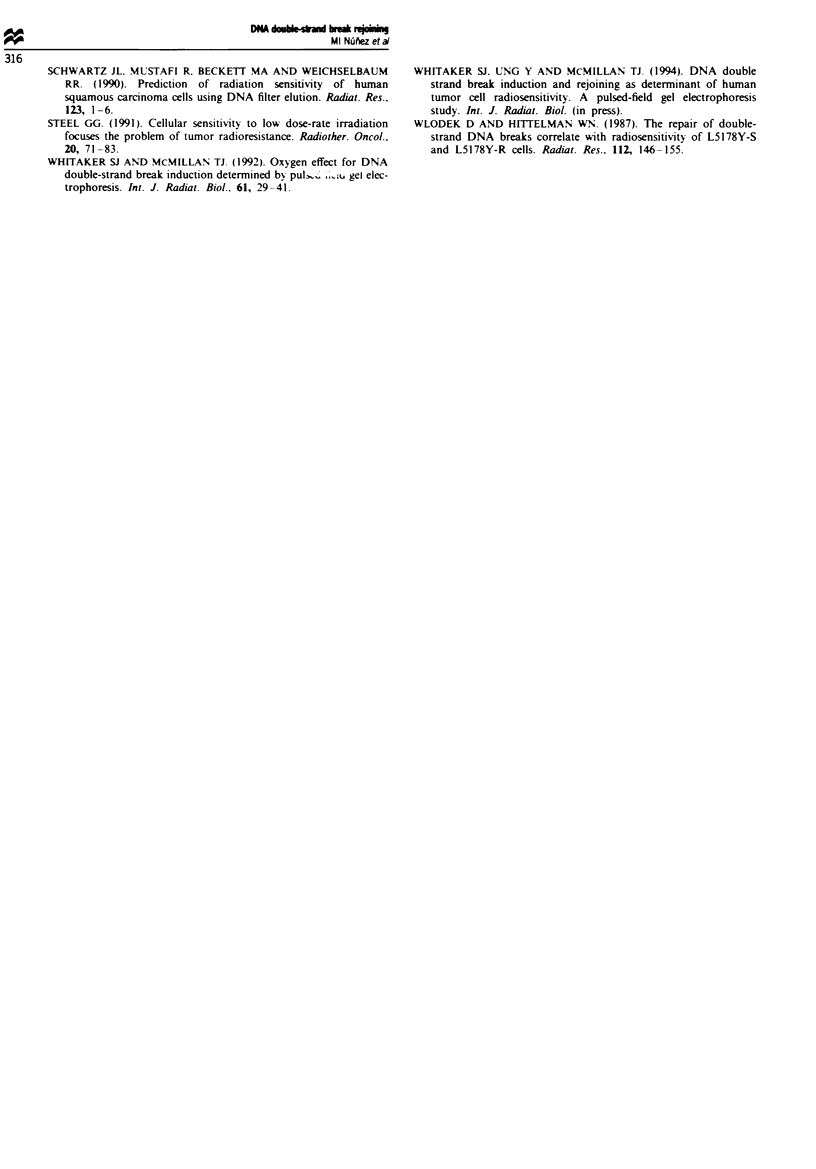# Radiation-induced DNA double-strand break rejoining in human tumour cells.

**DOI:** 10.1038/bjc.1995.62

**Published:** 1995-02

**Authors:** M. I. Núñez, M. Villalobos, N. Olea, M. T. Valenzuela, V. Pedraza, T. J. McMillan, J. M. Ruiz de Almodóvar

**Affiliations:** Departamento de Radiología y Medicina Física, Hospital Universitario, Facultad de Medicina, Granada, Spain.

## Abstract

Five established human breast cancer cell lines and one established human bladder cancer cell line of varying radiosensitivity have been used to determine whether the rejoining of DNA double-strand breaks (dsbs) shows a correlation with radiosensitivity. The kinetics of dsb rejoining was biphasic and both components proceeded exponentially with time. The half-time (t1/2) of rejoining ranged from 18.0 +/- 1.4 to 36.4 +/- 3.2 min (fast rejoining process) and from 1.5 +/- 0.2 to 5.1 +/- 0.2 h (slow rejoining process). We found a statistically significant relationship between the survival fraction at 2 Gy (SF2) and the t1/2 of the fast rejoining component (r = 0.949, P = 0.0039). Our results suggest that cell lines which show rapid rejoining are more radioresistant. These results support the view that, as well as the level of damage induction that we have reported previously, the repair process is a major determinant of cellular radiosensitivity. It is possible that the differences found in DNA dsb rejoining and the differences in DNA dsb induction are related by a common mechanism, e.g. conformation of chromatin in the cell.


© 1995 Stockton Press All rights reserved 0007-0920/95 $9.00

### Radiation-induced DNA double-strand break rejoining in human tumour cells

MI Núñez[1], M Villalobos[1], N Olea[1], MT Valenzuela[1], V Pedraza[1], TJ McMillan[2] and JM Ruiz de Almodóvar[1]

[1]*Laboratorio de Investigaciones Médicas y Biología Tumoral, Departamento de Radiología y Medicina Física, Hospital Universitario, Facultad de Medicina, 18071 Granada, Spain;* [2]*Radiotherapy Research Unit, The Institute of Cancer Research, 15 Cotswold Road, Sutton, Surrey SM2 5NG, UK.*

**Summary** Five established human breast cancer cell lines and one established human bladder cancer cell line of varying radiosensitivity have been used to determine whether the rejoining of DNA double-strand breaks (dsbs) shows a correlation with radiosensitivity. The kinetics of dsb rejoining was biphasic and both components proceeded exponentially with time. The half-time ($t_{1/2}$) of rejoining ranged from 18.0 ± 1.4 to 36.4 ± 3.2 min (fast rejoining process) and from 1.5 ± 0.2 to 5.1 ± 0.2 h (slow rejoining process). We found a statistically significant relationship between the survival fraction at 2 Gy (SF2) and the $t_{1/2}$ of the fast rejoining component ($r = 0.949$, $P = 0.0039$). Our results suggest that cell lines which show rapid rejoining are more radioresistant. These results support the view that, as well as the level of damage induction that we have reported previously, the repair process is a major determinant of cellular radiosensitivity. It is possible that the differences found in DNA dsb rejoining and the differences in DNA dsb induction are related by a common mechanism, e.g. conformation of chromatin in the cell.

**Keywords:** radiosensitivity; DNA double-strand breaks; dsb rejoining; pulsed field gel electrophoresis

The repair of radiation-induced lesions in DNA is widely believed to be a major determinant of the degree of radiosensitivity in mammalian cells. Evidence for this largely comes from radiosensitive mutants in which clear defects in the rejoining of DNA double-strand breaks (dsbs) have been detected (Kemp *et al.*, 1984; Iliakis *et al.*, 1992). The *xrs* radiosensitive mutants of Chinese hamster ovary (CHO) cells, for example, exhibit a much reduced overall capacity for repair of dsbs, although there is some debate as to whether the rate of dsb rejoining is altered in these cells (Dahm-Daphi *et al.*, 1993). Apart from these extreme cases, the relationship between various repair parameters is less clear-cut. Of the many dsbs that are produced in DNA by ionising radiation, most are repaired within a few hours at 37°C (Wlodek and Hittelman, 1987; Schwartz *et al.*, 1988). Several studies have investigated the influence of the rate and extent of rejoining in cell lines differing in sensitivity, but no clear pattern has emerged (Schwartz *et al.*, 1988, 1990).

In a group of six human tumour cell lines we have previously proposed that the observed variation in initial dsbs seen immediately after irradiation does correlate with radiosensitivity, although an analysis of fragment sizes is necessary (Ruiz de Almodóvar *et al.*, 1994*a*). It was the aim of this study to examine whether these cell lines also differ in their ability to remove dsbs following a single dose of radiation.

### Materials and methods

### *Cell lines*

Six human tumour cell lines were studied in this work. The origins and some characteristics of these were given in a previous paper (Ruiz de Almodóvar *et al.*, 1994). Five of the cell lines were derived from breast cancers (MCF-7 clones BUS and BB, T47D clones B1 and B8 and EVSA-T) and one from a bladder carcinoma (RT112). Cell cultures were grown in Dulbecco's modified Eagle medium (DMEM, Gibco), supplemented with 10% fetal calf serum (FCS). All media contained, in addition, penicillin (100 units ml$^{-1}$) and streptomycin (0.1 mg ml$^{-1}$). Cells were incubated at 37°C in plastic cell culture flask (Nunc) in 5% carbon dioxide in air. Freedom from mycoplasma contamination was checked periodically by testing with Hoescht 33528. Experiments were conducted on cells in exponential growth phase maintained by passage twice a week.

### *Irradiation*

Irradiation of cells was performed using a 10 TBq $^{60}$Co source at a dose rate of 1-2 Gy min$^{-1}$ for cell survival and 5 Gy min$^{-1}$ for dsb rejoining measurements. Dose rate was determined by a Victoreen 500 with a Nuclear Enterprise model 23332 ionisation chamber.

### *Clonogenic assays*

Acute-dose clonogenic assays were performed in monolayer culture as previously described (Ruiz de Almodóvar *et al.*, 1994). Briefly, graded inocula of test and control cells were seeded in triplicate into 25 cm$^2$ plastic tissue culture flasks. Irradiations were performed after 4 h when cells were attached. Cell colonies of > 50 cells were counted after 14-21 days and survival data were fitted using the linear-quadratic model [$\text{LnSF} = -(\alpha D + \beta D^2)$]. Parameters $\alpha$ and $\beta$ were determined by non-linear regression analysis. Three experiments were performed on each cell line.

### *DNA dsb rejoining*

Cells in exponential growth were radiolabelled with [$^{14}$C]thymidine (1.8 kBq ml$^{-1}$) for 48 h followed by 18-24 h chase in unlablled medium. After replacement of medium with cold (0°C) complete medium, test flasks were irradiated with 45 Gy while still attached in monolayer. The irradiation was performed at 0°C on ice. Following irradiation the 'cold' medium was replaced by 37°C DMEM + 10% FCS and cells were incubated for 0, 5, 15, 30, 45, 60, 75 and 90 min and 2, 4, 6, 8, 10 and 24 h. DNA rejoining was stopped by addition of excess ice-cold medium and transfer of the flasks to 4°C. We have looked carefully at the influence of this temperature on pulsed-field gel electrophoresis (PFGE) and there is no effect over 24 h. After 24 h, all the test and control flasks were harvested by mechanical disaggregation. Cells were

Correspondence: JM Ruiz de Almodóvar
Received 5 April 1994; revised 7 July and 12 September 1994; accepted 30 September 1994

washed and centrifuged at 4°C and mixed with ultra-low gelling temperature agarose (type IX, Sigma) at 15°C and a cell concentration of $1.2 \times 10^6$ cells $ml^{-1}$. The cell-agarose suspension was pipetted into moulds and cell plugs formed and were kept at 4°C for 1 h. These cell plugs were transferred into 30 ml plastic universal tubes containing ice-cold lysis buffer, pH 7.6, comprising 2% sodium lauryl sarkosine, 0.5 M EDTA (both Sigma) and 0.5 mg $ml^{-1}$ Proteinase K (Boehringer-Mannheim). Lysis proceeded on ice for 1 h then at 37°C for 24 h.

To determine whether the level of dose administrated has any influence on the DNA dsb kinetic rejoining process, we have also performed several experiments using two cell lines with clear differences in radiosensitivity (MCF-7 BUS, surviving fraction at 2 Gy, SF2 = 33%; and EVSA-T, SF2 = 65%). Cells were irradiated at 15 and 30 Gy and two experiments were performed on each cell line.

*Pulsed-field gel electrophoresis*

DNA dsb measurements were performed on a clamped homogeneous electric field pulse-field unit (CHEF-DR-II, Bio-Rad) as described previously (Whitaker and McMillan, 1992). Sections of 25 µl of each cell plug containing the DNA from approximately $10^5$ cells were loaded into the wells of a 0.8% agarose gel (Type V, Sigma) and subjected to CHEF-PFGE at 45 V, with field switching interval of 60 min for a total run time of 96 h. Electrophoresis buffer was 0.1 M Tris, 0.1 M borate and 0.2 mM EDTA (Sigma), pH 8.4. DNA size markers were included in each gel (*Saccharomyces cervisiae* and *Schizosaccharomyces pombe*, both from Bio-Rad). Following electrophoresis the gels were stained with ethidium bromide (0.5 µg $ml^{-1}$) then washed and, under UV transillumination, each lane of the gel was separated from its well and cut into 5 mm sections. Gel pieces were heated in 100 µl of 1 M hydrochloric acid and, when melted, neutralised with 100 µl of 1 M sodium hydroxide and mixed with scintillation fluid (Optiphase Type II, LKB). Isotope activity was determined on a 2800 LS Beckman liquid scintillation counter. At least two experiments were performed for each cell line.

*Assessment of DNA dsb rejoining kinetics*

The fraction of activity released (FAR) was calculated from the ratio of $^{14}C$ activity detected from all the sections in the lane to the total activity, i.e.

$$FAR = \frac{d.p.m._{lane}}{d.p.m._{lane} + d.p.m._{well}}$$

The values of the fraction of damage remaining (FDR) were plotted against the post-irradiation incubation time (Figure 1), where $FDR = FAR_t/FAR_0$.

After subtraction of the FDR value at 24 h incubation, the rejoining of DNA dsbs was assessed as the ratio between the FDR values obtained from each time point, $FDR_t$, and the FDR value corresponding to time $t = 0$.

$$RR = \frac{FDR_t}{FDR_0}$$

where RR is the rejoining ratio.

The RR values were plotted against time in semilogarithmic coordinates.

The kinetics of dsb rejoining during post-irradiation incubation of cells was fitted to a model based on two unsaturated, or first-order, reactions and can be described by the following equation (Frankenberg-Schwager and Frankenberg, 1992).

$$RR = \frac{FDR_t}{FDR_0} = f_1 \times \exp(-k_1 t) + f_2 \times \exp(-k_2 t)$$

where $f_1$ and $f_2$ are the fractions of initial dsbs being rejoined by the first and the second component of the biphasic kinetics and $k_1$ and $k_2$ are the corresponding rate constants of first-order rejoining components.

The final part of the rejoining curve was fitted by linear regression. The value on the regression line for the second component was then subtracted from the RR at the early time points prior to fitting these points by linear regression (Figure 2).

In calculating the kinetics of rejoining by this method we have assumed a linear relationship between dsbs and FAR. While the induction curves for these lines are not strictly linear for these cell lines (Ruiz de Almodóvar *et al.*, 1994) the analysis of the induction data as fitted by a linear relationship (Table I) is still good and the conversion of the data to Gy equivalents on the basis of the damage induction curves does not influence our conclusions from these data.

### Results

*Clonogenic survival assay*

The acute dose clonogenic survival curves were fitted by the linear-quadratic expression. The $\alpha$ and $\beta$ parameters and values of the survival fraction at 2 Gy (SF2) are shown in Table II.

*DNA dsb rejoining kinetics*

Figure 1 shows the kinetics of dsb rejoining of the six human tumour cell lines studied. The number of dsbs that remained unrejoined after a 24 h incubation period was subtracted from the number of dsbs measured after the other repair times. In this way the kinetics of only the rejoined dsbs was obtained (Frankenberg-Schwager and Frankenberg, 1992; Frankenberg-Schwager *et al.*, 1990) (Figure 2). It is apparent that the kinetic of dsb rejoining is biphasic and proceeds exponentially with time for both components. Tables III and IV summarise the values of the parameters $f_1$ and $t_{1/2}$ (fast component) and $f_2$ and $t_{1/2}$ (slow component) of the biphasic kinetics for each tumour cell line used. The half-time of rejoining ($t_{1/2}$) corresponding to each component was calculated from $t_{1/2} = -\ln 2/k$. These values ranged from 18.0 ± 1.4 to 36.4 ± 3.2 min (fast rejoining process) and from 1.5 ± 0.2 to 5.1 ± 0.2 h (slow rejoining process).

*Relationship between the rate of rejoining dsb and radiosensitivity*

Figure 3 shows the relationship between the rates of two components of rejoining and radiosensitivity (SF2). In each case the data have been fitted by linear regression, and in the case of the fast component (Figure 3a) there is a significant correlation between the two parameters ($SF2 = -0.017\ t_{1/2} + 1.1$; $r = 0.949$, $P = 0.0039$), but with the slow component the correlation does not reach significance $P = 0.15$) (Figure 3b). Although we have no reason to do so, if data for one of the cell lines studied (EVSA-T) are discarded then a significant relationship between SF2 and the rate of the slow component is seen. Overall, we therefore conclude that radioresistant cells have a faster half-time of rejoining than sensitive cells.

We found no relationship between the *f*-values and the cellular radiosensitivity parameters.

*Dose-effect*

Figure 4 shows the dose-effect results found in both MCF-7 BUS and EVSA-T cell lines. The half-times of the fast component of rejoining kinetic process appear to be dose independent. Actually the values obtained for 15 Gy and 30 Gy in both cell lines are very close to those found for 45 Gy.

### Discussion

The loss of proliferative capacity in irradiated cells is believed to depend on:

(a) *The number of initial radiation-induced DNA dsbs* (Radford, 1985; Peacock *et al.*, 1992; Whitaker and McMil-

**Figure 1** DNA dsb rejoining curves for six human tumour cell lines as the decrease in the fraction of damage remaining (FDR) following irradiation with 45 Gy and varying repair times at 37°C. Data points represent the means (and s.e.m.) of three experiments (only two for MCF-7 BB). The dotted line represents the fraction of irreparable damage (value of FDR after 24 h incubation).

**Figure 2** Kinetics of rejoinable dsbs is expressed as a ratio (RR). The experimental points have been fitted to two-component unsaturated (first-order) rejoining process.

**Table I** Parameters of initial radiation-induced DNA damage

| *Cell line* | *Slope*[a] | r | P |
|---|---|---|---|
| MCF-7 BUS | − 0.83 ± 0.10 | − 0.944 | <0.0001 |
| MCF-7 BB | − 0.75 ± 0.09 | − 0.943 | <0.0001 |
| T47D-B1 | − 1.27 ± 0.12 | − 0.966 | <0.0001 |
| T47D-B8 | − 0.95 ± 0.05 | − 0.989 | <0.0001 |
| EVSA-T | − 1.00 ± 0.07 | − 0.960 | <0.0001 |
| RT-112 | − 1.06 ± 0.19 | − 0.914 | <0.0015 |

[a]Slope of DNA damage induction curve from Ruiz de Almodóvar *et al.* (1994). *r*, correlation coefficient; *P*, *P*-value. Figures are means ± s.e.m. from five observations.

**Table II** Parameters of acute dose-survival curves

| *Cell line* | $\alpha$ *(Gy)* | $\beta$ *(Gy$^{-2}$)* | *SF2* |
|---|---|---|---|
| MCF-7 BUS | 0.536 ± 0.038 | 0.021 ± 0.004 | 32.9 ± 3.7 |
| MCF-7 BB | 0.316 ± 0.019 | 0.023 ± 0.006 | 50.2 ± 2.3 |
| T47D-B1 | 0.206 ± 0.024 | 0.036 ± 0.002 | 58.5 ± 3.1 |
| T47D-B8 | 0.190 ± 0.015 | 0.040 ± 0.001 | 54.9 ± 1.3 |
| EVSA-T | 0.260 ± 0.051 | 0.016 ± 0.005 | 64.7 ± 3.2 |
| RT-112 | 0.120 ± 0.018 | 0.040 ± 0.002 | 67.7 ± 1.5 |

Clonogenic cell survival data were fitted to the linear quadratic equation. SF2, surviving fraction at 2 Gy. Figures are means ± s.e.m.

**Table III** Parameters of DNA dsb rejoining kinetics

| Cell line | $f_1$ | Fast component $(t_{1/2})_1$ (min) | r | P |
|---|---|---|---|---|
| MCF-7 BUS | 0.70 | 36.9 ± 3.2 | −0.973 | <0.001 |
| MCF-7 BB | 0.61 | 32.6 ± 3.3 | −0.970 | <0.001 |
| T47D-B1 | 0.72 | 27.5 ± 3.3 | −0.956 | <0.001 |
| T47D-B8 | 0.62 | 28.1 ± 1.7 | −0.986 | <0.001 |
| EVSA-T | 0.62 | 22.3 ± 2.0 | −0.977 | <0.001 |
| RT-112 | 0.83 | 18.0 ± 1.4 | −0.989 | <0.001 |

$f_1$, fraction of the initial double-strand break being rejoined by the fast component; $(t_{1/2})_1$, half-time of the fast component of the rejoining kinetics; *r*, correlation coefficient; *P*, *P*-value. Figures are means ± s.e.m.

**Table IV** Parameters of DNA dsb rejoining kinetics

| Cell line | $f_2$ | Slow component $(t_{1/2})_2$ (hours) | r | P | Residual damage |
|---|---|---|---|---|---|
| MCF-7 BUS | 0.30 | 5.1 ± 0.2 | −0.997 | <0.01 | 5.7 ± 0.8 |
| MCF-7 BB | 0.39 | 2.4 ± 0.1 | −0.998 | <0.01 | 7.3 ± 1.4 |
| T47D-B1 | 0.28 | 2.8 ± 0.1 | −0.992 | <0.001 | 10.5 ± 2.1 |
| T47D-B8 | 0.38 | 2.8 ± 0.1 | −0.996 | <0.01 | 8.1 ± 4.6 |
| EVSA-T | 0.38 | 4.1 ± 0.1 | −0.990 | <0.01 | 7.2 ± 3.3 |
| RT-112 | 0.17 | 1.5 ± 0.2 | −0.990 | <0.01 | 10.2 ± 3.0 |

$f_2$, fraction of the initial double-strand break being rejoined by the slow component. $(t_{1/2})_2$, half-time of the slow component of the rejoining kinetics. r, correlation coefficient; P, *P*-value; Residual damage, percentage of activity released at 24 h corrected for extraction from non-irradiated controls. Figures are means ± s.e.m.

lan, 1992). We have recently studied the relationship between this end point and the parameters obtained from the acute-dose survival curve, using the same set of cell lines that have been used in this work. Our data support the view that the initial damage is a major determinant of intrinsic cell radiosensitivity (Ruiz de Almodóvar *et al*., 1994).

(b) *The number of unrejoined DNA dsbs* (Blöcher *et al*., 1991; Dahm-Daphi *et al*., 1993). It has generally been found DNA dsb rejoining is complete within 15 h after irradiation, and often sooner than this (Dahm-Daphi *et al*., 1993). We therefore considered the FAR values obtained for 24 h after subtracting the FAR values obtained from control flasks as our measure of residual damage (Table IV). Our results show that there is no relationship between residual damage and SF2. This result agrees with our previous experiments in RT-112 bladder carcinoma cell line, in which we show that the differences in the clonogenic cell survival at different dose rates cannot be explained by the final level of unrejoined dsbs (Ruiz de Almodóvar *et al*., 1994) and it is supported by other studies in which a comparison of cells with different sensitivity has shown no differences in unrejoined dsbs (Koval and Kazmar, 1988). This result could be a reflection of inadequate sensitivity of PFGE at the doses used since correlations between sensitivity and residual damage have been found in studies in which higher doses were used (Blöcher *et al*., 1991; R. Wurm, personal communication). Alternatively we believe it could be due to one of the main problems with any physical measure of DNA integrity: the inability to distinguish between repair and misrepair (Ruiz de Almodóvar *et al*., 1994).

(c) *The rate of rejoining of DNA dsbs*. Following damage induction, numerous processes remove and repair the damage in an attempt to restore the genetic sequence to its original state. DNA damage may be correctly repaired, repaired incorrectly (misrepaired) or completely unrepaired.

Our results show that the DNA dsb rejoining process follows biphasic kinetics with a rapid initial rate followed by

**Figure 3** (**a**) Relationship between SF2 and $t_{1/2}$ of the fast rejoining component. (**b**) Relationship between SF2 and $t_{1/2}$ of the slow rejoining component.

**Figure 4** Half-times of the fast rejoining component at different doses. ▨, MCF-7 BUS; □, EVSA-T.

a much slower second component. This has been noted previously (Bryant *et al*., 1984; Iliakis *et al*., 1990) and it has been termed a two-component/unsaturated/dose-dependent process (TDU) (Frankenberg-Schwager *et al*., 1990). The two components of rejoining kinetic show a dose-independent rate of dsb rejoining, however the dose dependency of the proportions of the two components slow down the overall rate of dsb rejoining with increasing dose (Frankenberg-Schwager and Frankenberg, 1992).

It is possible that the multiple phases are due to the repair of different types of lesion, with the residual damage as a final subset of lesions (Steel, 1991). However, this is untestable with current technology. It has also been suggested that the initial fast component entails rejoining of dsbs by enzymes which are constitutively expressed in mammalian cells (Hittelman and Pollard, 1982; Radford, 1987; Boothman *et al.*, 1989). The late phase, which proceeds at a significantly slower rate, is characteristically blocked by inhibitors of protein, RNA and DNA synthesis (Iliakis, 1989) suggesting that it may require the induction of specific genes and gene products to repair more complex types of DNA lesions (Boothman *et al.*, 1989; Haimovich-Friedman *et al.*, 1991). We suggest that misrepair may occur in both fast and slow rejoining components, but it must be more probable when the rejoining kinetic is slower. What is still unknown is how DNA packaging in cells affects DNA repair. Structural differences (euchromatin or heterochromatin, for example) may alter the nature of the damage, the function and the positioning of repair systems.

Our results show a close relationship between cell survival and the half-time of dsb rejoining corresponding to fast components. A survey of the literature reveals that our results agree with those published by Kelland *et al.* (1988), Schwartz *et al.* (1990) and Giaccia *et al.* (1992). Studies on other cell lines have also suggested a relationship between the slow component of rejoining process and cell radiosensitivity (Whitaker *et al.*, 1994). Thus, cells with a rapid rate of rejoining are generally more resistant to ionising radiation, perhaps because of a higher fidelity of rejoining.

Overall, our view is that sensitive cells suffer more dsbs per dose unit. In addition, the process of dsb rejoining is slower than in the radioresistant ones. It is possible that these two observations are directly linked, i.e. that the induction of more damage leads to a slower rate of repair because of the greater strain on the repair systems, although we have evidence for a dose independence of repair rates within two different cell lines (Figure 4). Alternatively, they may be indirectly linked through a common cause such as the conformation of DNA (Oleinick *et al.*, 1984; Patil *et al.*, 1985; Barendsen, 1988; Olive, 1992).

**Acknowledgements**
This work was supported by CICYT SAL 89-1115 and DGICYT HB-250, Spain. Grants PN90 26206833 to MIN and 92 8837784 to MTV. Grants from the British Council greatly aided this collaboration. TJM is supported by the Cancer Research Campaign and the Medical Research Council.

### References

BARENDSEN GW. (1988). Radiation-induced DNA damage in relation to linear and quadratic terms of dose-effect relationships for cell reproductive death. *Br. J. Radiol.*, **24** (Suppl.), 53-56.

BLOCHER D, SIGUT D AND HANNAN MA. (1991). Fibroblasts from ataxia-telangiectasia (AT) and AT heterozygotes show an enhanced level of residual DNA double strand breaks after low dose-rate irradiation as assayed by pulsed field gel electrophoresis. *Int. J. Radiat. Biol.*, **60**, 791-802.

BOOTHMAN DA, BOUVARD I AND HUGHES EN. (1989). Identification and characterization of X-ray induced proteins in human cells. *Cancer Res.*, **49**, 2871-2878.

BRYANT PE, WARRING R AND AHNSTROM G. (1984). DNA repair kinetics after low doses of X-rays. A comparison of results obtained by the unwinding and nucleotide sedimentation methods. *Mutat. Res.*, **131**, 19-26.

DAHM-DAPHI J, DIKOMEY E, PYTTLIK C AND JEGGO PA. (1993). Reparable and non-reparable DNA strand breaks induced by X-irradiation in CHO K1 cells and the radiosensitive mutants xrs-1 and xrs-5. *Int. J. Radiat. Biol.*, **64**, 19-26.

FRANKENBERG-SCHWAGER M AND FRANKENBERG D. (1992). Shouldered survival curves in accordance with the unsaturated rejoining kinetics of DNA double-strand breaks. *Br. J. Radiol.*, **24** (Suppl.), 23-27.

FRANKENBERG-SCHWAGER M, FRANKENBERG D, HARBICH R AND ADAMCZYK C. (1990). A comparative study of rejoining of DNA double-strand breaks in yeast irradiated with 3.5 MeV α-particles or with 30 MeV electrons. *Int. J. Radiat. Biol.*, **57**, 1151-1168.

GIACCIA AJ, SCHWARTZ J, SHIEH J AND BROWN JM. (1992). The use of asymmetric-field inversion gel electrophoresis to predict tumor cell radiosensitivity. *Radiother. Oncol.*, **24**, 231-238.

HAIMOVITZ-FRIEDMAN A, VLODAVSKY I, CHAUDHURI A, WITTE L AND FUKS Z. (1991). Autocrine effects of fibroblast growth factor in repair of radiation damage in endothelial cells. *Cancer Res.*, **51**, 2552-2558.

HITTELMAN WN AND POLLARD M. (1982). A comparison of the DNA and chromosome repair kinetics after irradiation. *Radiat. Res.*, **92**, 497-509.

ILIAKIS G. (1989). Radiation-induced potentially lethal damage: DNA lesion supceptibles to fixation. *Int. J. Radiat. Biol.*, **53**, 541-584.

ILIAKIS G, METZGER L, MUSCHEL RV AND MCKENNA WG. (1990). Induction and repair of DNA double strand breaks in radiation-resistant cells obtained by transformation of primary rat embryo cells with the oncogenes H-ras and v-myc. *Cancer Res.*, **50**, 6575-6579.

ILIAKIS G, METHA R AND JACKSON M. (1992). Level of DNA double-strand break rejoining in chinese hamster xrs-5 cells is dose-dependent: implications for the mechanism of radiosensitivity. *Int. J. Radiat. Biol.*, **61**, 315-321.

KELLAND LR, EDWARDS SM AND STEEL GG. (1988). Induction and rejoining of DNA double-strand breaks in human cervix carcinoma cell lines of differing radiosensitivity. *Radiat. Res.*, **116**, 526-538.

KEMP LM, SEGDWICK SG AND JEGGO PA. (1984). X-ray sensitive mutants of chinese hamster ovary cells defective in double-strand break rejoining. *Mutat. Res.*, **132**, 189-196.

KOVAL TM AND KAZMAR ER. (1988). DNA double-strand break repair in eukaryotic cell lines having radically different radiosensitivities. *Radiat. Res.*, **113**, 268-277.

PATIL MS, LOCHER SE AND HARIHAN PV. (1985). Radiation induced thymine base damage and its excision repair in active and inactive chromatin of HeLa cells. *Int. J. Radiat. Biol.*, **48**, 691-700.

OLEINICK NL, CHIU S AND FRIEDMAN LR. (1984). Gamma irradiation as a probe of chromatin structure: damage and repair of chromatin in the metasphase chromosome. *Radiat. Res.*, **98**, 629-641.

OLIVE PL. (1992). DNA organization affects cellular radiosensitivity and detection of initial DNA strand breaks. *Int. J. Radiat. Biol.*, **62**, 389-396.

PEACOCK JH, EADY JJ, EDWARDS SM, MCMILLAN TJ AND STEEL GG. (1992). The intrinsic α/β ratio for tumor cells: is it a constant? *Int. J. Radiat. Biol.*, **61**, 479-487.

RADFORD IR. (1985). The level of induced double strand breakage correlate, with cell killing after X-irradiation. *Int. J. Radiat. Biol.*, **48**, 45-54.

RADFORD IR. (1987). Effect of cell-cycle position and dose on the kinetics of DNA double strand breakage repair in X-irradiated chinese hamster cells. *Int. J. Radiat. Biol.*, **52**, 555-563.

RUIZ DE ALMODÓVAR JM, NÚÑEZ MI, MCMILLAN TJ, OLEA N, MORT C, VILLALOBOS M, PEDRAZA V AND STEEL GG. (1994). Initial DNA damage is a determinant of intrinsic cellular radiosensitivity. *Br. J. Cancer*, **69**, 457-462.

RUIZ DE ALMODÓVAR JM, BUSH C, PEACOCK JH, STEEL GG, WHITAKKER SJ AND MCMILLAN TJ. (1994). Dose-rate effect for DNA damage induced by ionizing radiation in human tumor cells. *Radiat. Res.*, **138**, S93-S96.

SCHWARTZ JL, ROTMENSCH J, GIOVANAZZI S, COHEN MB AND WEICHSELBAUM RR. (1988). Faster repair of DNA double-strand breaks in radioresistant human tumor cells. *Int. J. Radiat. Oncol. Biol. Phys.*, **15**, 907-912.

SCHWARTZ JL, MUSTAFI R, BECKETT MA AND WEICHSELBAUM RR. (1990). Prediction of radiation sensitivity of human squamous carcinoma cells using DNA filter elution. *Radiat. Res.*, **123**, 1-6.

STEEL GG. (1991). Cellular sensitivity to low dose-rate irradiation focuses the problem of tumor radioresistance. *Radiother. Oncol.*, **20**, 71-83.

WHITAKER SJ AND MCMILLAN TJ. (1992). Oxygen effect for DNA double-strand break induction determined by pulsed field gel electrophoresis. *Int. J. Radiat. Biol.*, **61**, 29-41.

WHITAKER SJ, UNG Y AND MCMILLAN TJ. (1994). DNA double strand break induction and rejoining as determinant of human tumor cell radiosensitivity. A pulsed-field gel electrophoresis study. *Int. J. Radiat. Biol.* (in press).

WLODEK D AND HITTELMAN WN. (1987). The repair of double-strand DNA breaks correlate with radiosensitivity of L5178Y-S and L5178Y-R cells. *Radiat. Res.*, **112**, 146-155.